# Severe chemosis and treatment following fronto-orbital advancement surgery for Crouzon syndrome: A case report

**DOI:** 10.1097/MD.0000000000024693

**Published:** 2021-02-19

**Authors:** Shui-Hua Wu, Tian-Jia Liu, Shuang-Shi Fan, Zhao-Hui Chen, Xi-Lang Wang, Shuo Gu

**Affiliations:** a Department of Neurosurgery, Hunan Children's Hospital, Changsha, Hunan; b Department of Neurosurgery, Shanghai Jiaotong University School of Medicine, Shanghai Children's Medical Center, Shanghai; c Department of Ophthalmology, Hunan Children's Hospital, Changsha; d Department of Neurosurgery, The Maternal and Child Health Hospital of Hainan Province, Haikou, Hainan, China.

**Keywords:** case report, chemosis, Crouzon syndrome, fronto-orbital advancement

## Abstract

**Rationale:**

Crouzon syndrome is a craniofacial malformation caused by premature fusion of fibrous sutures in infants. It is one of the most common craniosynostosis syndromes, and surgery is the only effective treatment for correcting it. Postoperative complications such as encephalocele, infections, hematoma have been reported. We herein report a case of a 62-month-old boy with Crouzon syndrome who underwent fronto-orbital advancing osteotomy, cranial vault remolding, and extensive osteotomy and subsequently developed left proptosis and severe chemosis, these complications are rare and we believe it will be of use to clinicians, physicians, and researchers alike.

**Patient concerns:**

The patient's skull had been malformed since birth, and he had been experiencing paroxysmal headaches coupled with vomiting for 4 months. Having never received prior treatment, he underwent fronto-orbital advancement at our clinic; afterward, left proptosis and severe chemosis occurred.

**Diagnosis:**

The patient was diagnosed with Crouzon syndrome, and the complications included left proptosis and severe chemosis, confirmed by the clinical manifestations, physical examination, and computed tomography (CT).

**Intervention:**

We carried out cranial vault remodeling and fronto-orbital advancement. We applied ophthalmic chlortetracycline ointment on the conjunctivae, elevated the patient's head, evacuated the hematoma, and carried out a left blepharorrhaphy.

**Outcomes:**

The proptosis and chemosis resolved with no recurrence. No other complications occurred during the follow-up period (12 months), and CT scans revealed that the hematoma had disappeared. The calvarial vault reshaping was satisfactorily performed, and the patient's vision was not impaired.

**Lessons:**

Severe proptosis and chemosis are rare complications that can occur after fronto-orbital advancement for Crouzon syndrome. A detailed preoperative examination (including magnetic resonance imaging and CT) is essential for diagnosis. Complete hemostasis, evacuation of hematoma, and placement of a periorbital drainage tube during surgery all contribute to an effective treatment plan. An ophthalmic ointment should be administered, and the patient's head should be elevated during the procedure. Evacuation of retrobulbar epidural hematoma and blepharorrhaphy could also help relieve proptosis and chemosis. Our report describes 2 rare complications associated with the treatment for Crouzon syndrome, and we believe it will be of use to clinicians, physicians, and researchers alike.

## 1 Introduction

Crouzon syndrome is a craniofacial malformation caused by the premature fusion of fibrous sutures in infants and is one of the most common craniosynostosis syndromes. This autosomal dominant disorder has an incidence of 1 in 25,000 live births.^[1]^ Once diagnosed, further evaluation and early surgical management are warranted, with fronto-orbital advancement being the most effective treatment option. With an increase in the number of such cases, several postoperative complications have been reported. Caplan et al^[2]^ described an encephalocele developing as a late complication of cranial vault reconstruction in a patient with Crouzon syndrome. The patient benefited from the encephalocele repair, cranialization of the frontal sinus with bone grafting, and LeFort III osteotomies for mid-face advancement. Esparza and Hinojosa^[3]^ reported multiple intracranial complications among 296 cases, including wound infection, subgaleal hematoma, infected hematoma, empyema, dural tear, leakage of cerebrospinal fluid (CSF), basal encephalocele and plate scarring, and cerebral contusion. Extracranial complications included postoperative hyperthermia and infections of the urinary tract, central line, respiratory system, eyes, mechanical intravenous line, and viral infections. The complication most frequently reported was nonfiliated postoperative hyperthermia. Findings from other reports^[1–4]^ and our case suggest that the encephaloceles are complex defects that require an aggressive 2-team approach. Plastic surgeons and neurosurgeons reduce the herniated dura and perform direct repair, or duroplasty, and autologous calvarial or rib bone graft to repair the bony defect. Further challenges are the postsurgical complications of the respiratory tract, which could lead to apnea and death in extreme cases, such as a severe respiratory obstruction.

We herein report a case of a 62-month-old boy with Crouzon syndrome who underwent fronto-orbital advancing osteotomy, cranial vault remolding, and extensive osteotomy and developed unexpected left proptosis and severe chemosis occurred 3 days postsurgery. These complications posed a high risk of causing severe damage to the patient's vision. Although 2 similar cases have been reported before, some of the suggestions mentioned in these reports, such as “tight tracheostomy collar” and “lymphatic drainage being damaged,” are not applicable for our case. We believe that our report can help draft an efficient plan for the early diagnosis and effective treatment of these relatively rare complications after fronto-orbital advancement to treat Crouzon syndrome.

## 2 Case report

A 62-month-old boy was referred to our department for skull malformation and headaches. The child's skull was malformed since birth, as known by his parents. He experienced moderately severe paroxysmal headaches and vomiting for 4 months. He did not receive any treatment for these symptoms because there was no relevant medical history or family medical history. On physical examination, the patient presented turricephaly, brachycephaly, and a head circumference of 49.5 cm. Computed tomography (CT) and 3-dimensional reconstruction confirmed Crouzon syndrome accurately. The surgery to correct these malformations included fronto-orbital advancing osteotomy, remolding of the cranial vault, and extensive osteotomy. Finally, we reconstructed the prefused cranial suture. The surgery was successful.

**Figure 1:**
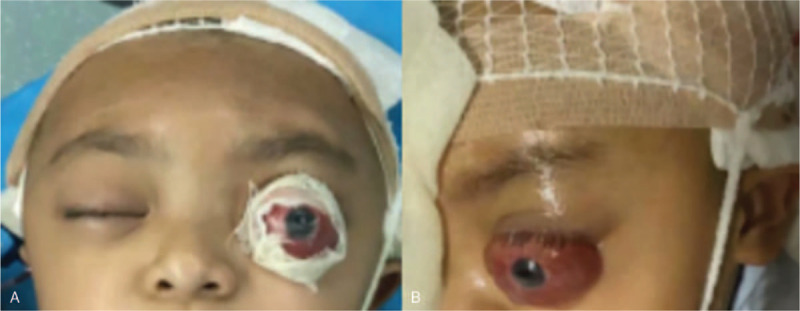
Proptosis and chemosis. Unexpected left proptosis and severe chemosis that occurred 3 days postsurgery. (A) Frontal view. (B) Lateral view.

**Figure 2:**
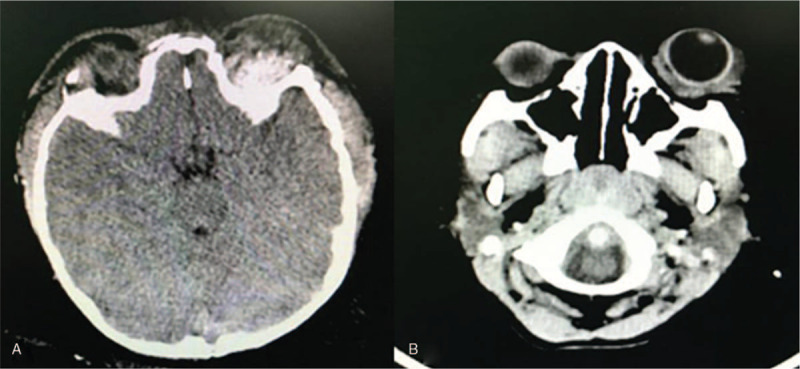
Cranial computed tomography scan after fronto-orbital advancement. (A) Retrobulbar epidural hematoma. (B) Left proptosis.

**Figure 3:**
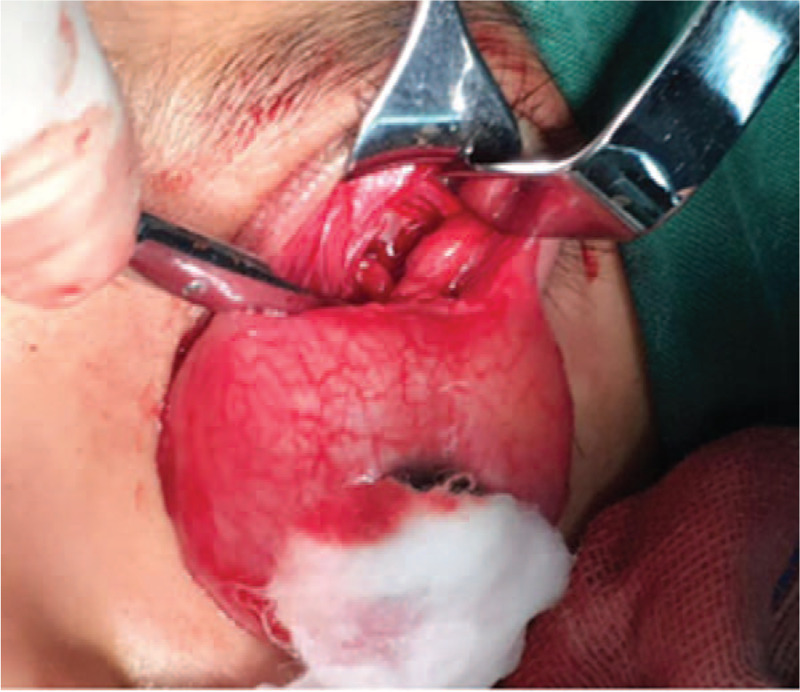
Evacuation of the hematoma. Incision of the left upper eyelids and orbital periosteum to evacuate the hematoma, performed on the 4th day after fronto-orbital advancement.

**Figure 4:**
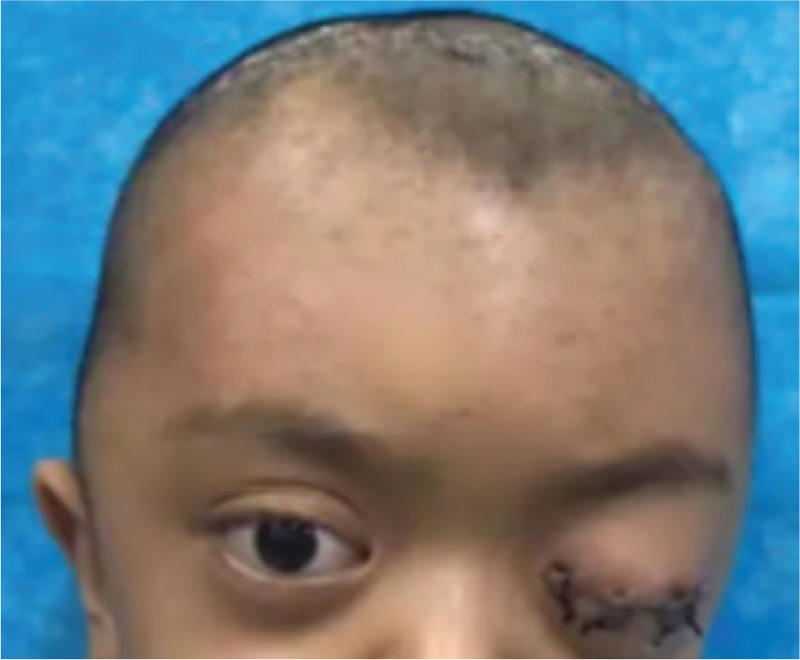
Six days after hematoma removal and left blepharorrhaphy. Chemosis and proptosis resolved gradually after evacuating the hematoma and performing a left blepharorrhaphy.

**Figure 5:**
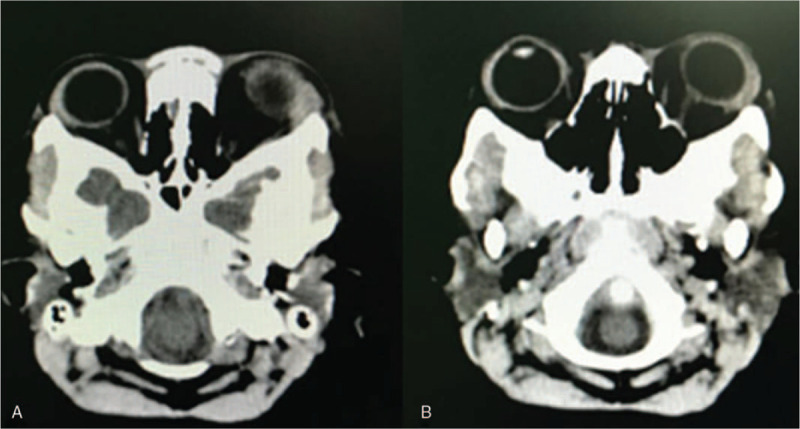
Postoperative computed tomography scan. (A) and (B) Complete resolution of the hematoma.

**Figure 6:**
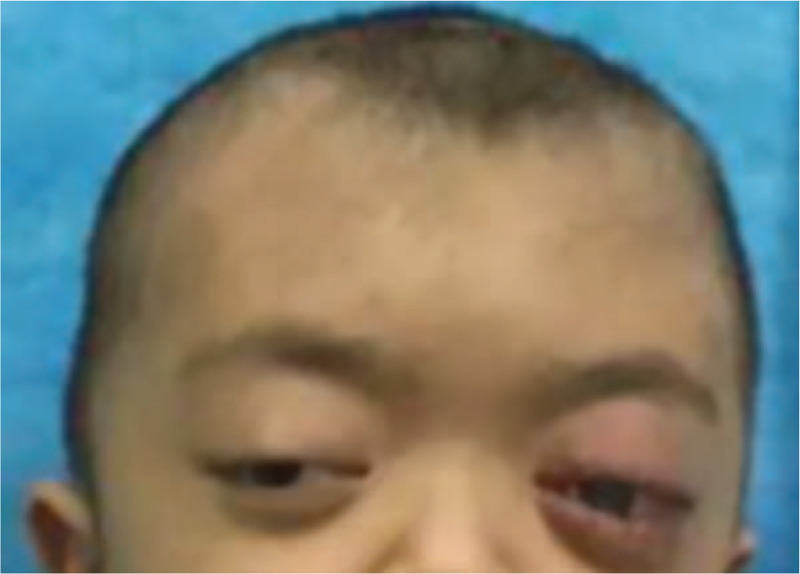
One month after hematoma removal and left blepharorrhaphy. The patient's proptosis and chemosis were completely resolved.

**Figure 7:**
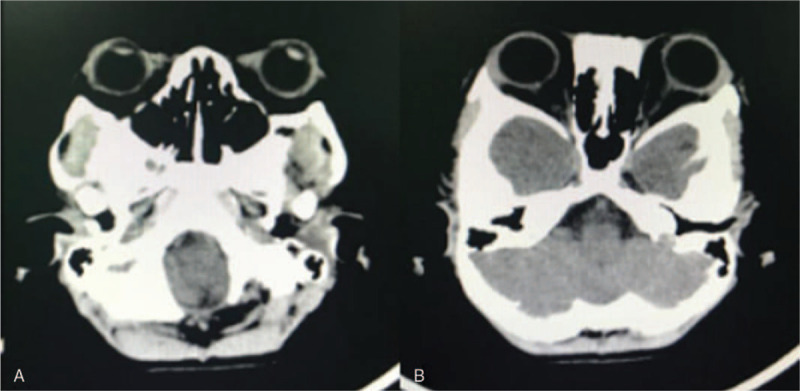
Postoperative computed tomography scans 1 year after hematoma removal and left blepharorrhaphy. (A) and (B) No hematoma recurrence.

However, unexpected left proptosis and severe chemosis occurred 3 days postsurgery (Fig. 1), which posed a high risk of causing severe damage to the patient's vision. The initial treatment of ophthalmic chlortetracycline (Aureomycin Jiangxi Xier Kangtai Pharmaceutical Co., Ltd, Ping Xiang City, China) ointment on the patient's conjunctivae twice a day and head elevation was insufficient to resolve this complication. A cranial CT scan revealed a retrobulbar epidural hematoma in the left eye (Fig. 2). Hence, we decided to incise the left upper eyelid and orbital periosteum on the 4th day postsurgery (Fig. 3). The hematoma was under the orbital periosteum and measured approximately 2 × 2 × 2 cm. The hematoma applied pressure on the left eyeball, occupied the orbital volume, and aggravated the exophthalmos. After hematoma evacuation and left blepharorrhaphy (Fig. 4), the chemosis and proptosis gradually resolved. The patient was discharged uneventfully 10 days after the remolding surgery and after receiving hemostasis and anti-infective agents as well as nutritional treatment. No other complications occurred during the follow-up period (12 months postsurgery), and CT scans confirmed that the hematoma disappeared completely (Fig. 5). At the age of 63 months, the patient showed no signs of proptosis or chemosis (Fig. 6), and at 74 months, the patient achieved satisfactory reshaping of the calvarial vault with no impairment to his vision. No recurrent hematomas were observed in the follow-up period (Fig. 7).

## 3 Discussion

Reconstruction of the cranial vault with fronto-orbital advancement is the key treatment strategy for craniosynostosis.^[5]^ Severe chemosis following fronto-orbital advancement has been reported previously by Laghmari et al^[5]^ and Hatef et al.^[6]^ At present, the etiology and standard treatment options for this complication are poorly understood.

We consulted experienced surgeons and the relevant literature to determine the causes of these complications. Hatef et al^[6]^ suggested that chemosis is caused by a combination of increased postoperative swelling and decreased effective drainage of the conjunctivae due to occlusion by the tight tracheostomy collar. Laghmari et al^[5]^ hypothesized that the cause might be orbital venous drainage disorder due to the deterioration of the transcalvarial emissary vein. Other potential causes include the impairment of the ophthalmic vein drainage due to the retrobulbar epidural hematoma occupying the orbital volume and aggravating the exophthalmos. Moreover, leakage of CSF into the orbit, after superior orbital wall fracture, and subsequent CSF accumulation in the superior conjunctival fornix may cause severe upper lid edema and chemosis.^[7]^ Osteotomy during surgery could also cause tears in the dura, leading to CSF leakage. Several studies have suggested that all types of chemosis are generally accompanied by inflammation at the outset and could result from trauma during surgery.^[8]^ Dellen formation could further stimulate a local inflammatory response and aggravate chemosis to form a positive “feedback” loop.^[8]^ Impairment of the eyelid and orbital lymphatic drainage could be another cause of chemosis^[8]^; however, this possible cause was excluded in our patient because we believed that the scalp coronoid incision and dissection did not damage the lymphatic drainage from the eye. This belief was supported and further strengthened by referring to findings from previously published studies and surgeries similar to ours.^[9–15]^

Although severe chemosis following fronto-orbital advancement is rare, we believe that it is helpful for surgeons to perform the following steps as part of an effective treatment plan for chemosis:

1. Assess cerebral and orbital venous drainage by magnetic resonance imaging and formulate preoperative designs for each patient while being cautious of the main emissary veins.^[5]^
2. Achieve complete hemostasis, evacuate hematomas (especially retrobulbar epidural hematoma), and place a periorbital drainage tube during surgery.
3. If proptosis and chemosis occur, an ophthalmic ointment should be applied to the conjunctiva and the patient's head should be elevated.
4. If the complications do not resolve, cranial CT scans should be performed to ascertain the cause.
5. Perform an evacuation of the retrobulbar epidural hematoma and blepharorrhaphy, as necessary, to relieve proptosis and chemosis.

In addition to the examples discussed above, Esparza and Hinojosa^[3]^ reviewed postoperative complications of craniosynostosis in 306 consecutive transcranial procedures. The most frequent complications observed were non-filiated postoperative hyperthermia (13.17% of the cases) followed by infections (8.10%), subcutaneous hematoma (6.08%), dural tears (5.06%), and CSF leakage (2.7%). Twenty five of these patients (5 Crouzon, 5 Apert, 3 Pfeiffer, 2 Saethre-Chotzen, and 10 non-syndromic multisutural craniosynostosis) underwent standard bilateral fronto-orbital advancement with expansive osteotomies. Complications included 1 wound infection, 2 dural tears, 2 persistent craniolacunae, and 1 subgaleal hematoma. Caplan et al^[2]^ reported a patient with Crouzon syndrome who had previously undergone forehead and cranial vault reconstruction as an infant and presented 22 years later with an encephalocele. Treatment included repair of the encephalocele, cranialization of the frontal sinus with bone grafting, and LeFort III osteotomies for mid-face advancement. The patient was satisfied with their appearance after the surgery.

## 4 Conclusions

We reported the case of a patient who developed severe chemosis following fronto-orbital advancement for Crouzon syndrome. Since the causes and required treatment for this complication are yet to be investigated and understood, we reported a successful treatment strategy performed on a patient with severe chemosis. We have also summarized previously published causes and treatments for chemosis following fronto-orbital advancement. Reviewing the complications following this surgery and drafting an efficient treatment strategy could help surgeons manage and treat them successfully when encountered.

Although we have successfully solved the complications at this time, some limitations related to the case remain: we should have performed the CT examination as soon as possible (regardless of the radiation risks) and asked for ophthalmic consultation, as visual impairment will bring great harm.

## Author contributions

**Conceptualization:** Shuo Gu.

**Data curation:** Tian-Jia Liu.

**Investigation:** Tian-Jia Liu.

**Methodology:** Shuo Gu.

**Project administration:** Zhao-Hui Chen.

**Resources:** Shui-Hua Wu.

**Software:** Shuang-Shi Fan.

**Supervision:** Xi-Lang Wang.

**Writing – original draft:** Tian-Jia Liu.

**Writing – review & editing:** Shui-Hua Wu.
